# Stem cell membrane-coated isotretinoin for acne treatment

**DOI:** 10.1186/s12951-020-00664-9

**Published:** 2020-07-28

**Authors:** Shiyi Wang, Rihua Jiang, Tianqi Meng, Fuqiang Zhang, Jing Li, Yongri Jin, JeungHoon Lee, Mingji Zhu, Jinlan Jiang

**Affiliations:** 1 Department of Dermatology, China-Japan Union Hospital of Jilin University, Changchun, Jilin China; 2 Scientific Research Center, China-Japan Union Hospital of Jilin University, Changchun, Jilin China; 3 College of Chemistry, Jilin University, Changchun, Jilin China; 4 Department of Dermatology, School of Medicine, Chungnam National University, Daejeon, Republic of Korea

**Keywords:** Stem cell, Acne, Isotretinoin, Transdermal, Nanoparticles

## Abstract

**Background:**

Topical isotretinoin is commonly used to treat acne. However, topical isotretinoin has side effects and can hardly permeate through the stratum corneum, the most important skin barrier. Therefore, this study aimed to demonstrate the efficacy of nanoparticles as stable carriers with great curative effects, low side effects, and strong transdermal ability.

**Results:**

In a rabbit model of hyperkeratinization, STCM-ATRA-NPs showed significant therapeutic efficacy. By contrast, negative therapeutic efficacy was observed in a golden hamster model of hyper sebum production. Scanning electron microscopy and Fourier transform infrared spectral analyses showed that nanoparticles could penetrate the stratum corneum. Western blotting demonstrated that the nanoparticles could enhance the transdermal efficacy of isotretinoin by reducing the effect of keratin and tight junction proteins. Further, nanoparticles enhanced endocytosis, thereby promoting drug penetration and absorption into the skin.

**Conclusion:**

STCM-ATRA-NPs were demonstrated to control isotretinoin release, reducing its side effects, and efficiently permeating through the skin by reducing the effect of keratin and tight junction proteins and enhancing endocytosis.
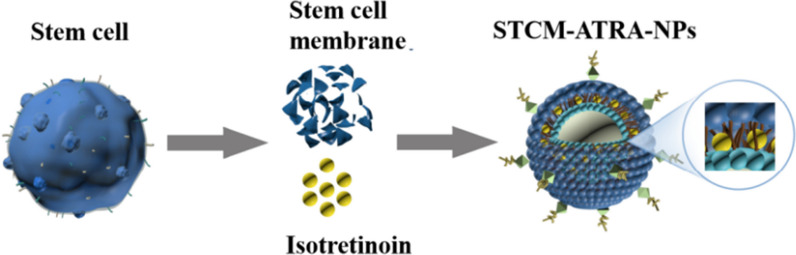

## Background

Acne is a chronic inflammatory disease and the most common dermatological disease [1], affecting about 85% teenagers worldwide, and it can persist into adulthood [2]. The disease can cause negative psychosocial consequences for the affected individual, including diminished self-esteem, social withdrawal due to embarrassment, depression, and unemployment, thus adequate therapy is needed [3]. There are four primary pathogenic factors resulting in acne, including sebum production, altered keratinization, inflammation, and bacterial keratinization. Currently, isotretinoin is the only drug that can target each of these factors [4, 5]. Topical external retinoids are also effective acne treatments [6]. However, topical application can lead to skin irritation such as erythema [7, 8].

Vesicles can increase isotretinoin concentration in the skin, decreasing its side effects [9]. Liposomes and chemical synthesis of isotretinoin, tretinoin, have been reported. Yet, liposomes and chemical materials are toxic and lack biological activity [10–12]. Currently, there are limited bio-membranes as vehicles for isotretinoin delivery and effective drug carriers to penetrate the main skin barrier, the stratum corneum (SC). Stem cells are a type of primitive undifferentiated cells with self-renewal and multi-directional differentiation potential, with low immunogenicity characteristics [13, 14]. Mesenchymal stem cells (MSCs) have been studied widely to elucidate their characteristics and unique properties as well as potential therapeutic applications in various diseases [15], including multiple clinical trials [16]. Many studies have used stem cell membranes (STCM) to conceal nanoparticles, showing biological characteristics such as long-term stability and drug-controlled release [17–19]. However, no study has investigated preparation of nanoparticles using STCM as a drug carrier to encapsulate small molecule drugs directly.

Transdermal delivery across the SC has garnered much attention [20]. Many approaches, including microneedling, have been attempted to improve transdermal delivery. Some studies have suggested that drug carriers (i.e., liposomes) could enhance drug penetration through fusing with SC lipids or by disturbing the intercorneocyte lipid organization [21, 22]. However, the mechanism of transdermal delivery has not been thoroughly investigated [22]. While most transdermal mechanisms focused on keratin, few studies have reported the role of tight junctions [23] and cell endocytosis [24].

In this study, the human umbilical cord mesenchymal stem cell (hMSC) membrane, as a natural liposome for drug delivery, was used to compose nanoparticles consisting of isotretinoin wrapped in the stem cell membrane (STCM-ATRA-NPs) to treat acne. Transmission electron microscopy (TEM), ultraviolet spectrophotometry, and dynamic light scattering (DLS) analyses were used to characterize STCM-ATRA-NPs. Designation of different preparation methods and ratio of stem cell membrane to isotretinoin were performed to find the appropriate encapsulation efficiency. Zeta potential was measured to investigate its stability. STCM-ATRA-NPs were shown to be small vesicles with a high encapsulation efficiency, showing stable and sustained drug release ability, less skin irritation, better treatment efficiency, and great transdermal ability. Further, we explored the transdermal mechanism by scanning electron microscopy (SEM) and Fourier transform infrared (FTIR) spectrometry to evaluate SC penetration. Finally, western blotting of endocytosis-related proteins, tight junction proteins, and keratin was conducted to investigate the mechanism of the transdermal and drug absorption pathways.

## Materials and methods

### Cell lines and antibodies

Human umbilical cord mesenchymal stem cell lines (hMSCs) were provided by the Scientific Research Centre of China-Japan Union Hospital, Jilin University (Changchun, China). Isotretinoin was purchased from APExBIO Technology LLC (Houston, TX, USA). Immunohistochemical antibodies against IL-8 and TNF-α were purchased from Bioscience Tec LLC (Tianjin, China). Antibodies against clathrin heavy chain were purchased from Abcam Tec (Shanghai, China). Antibodies against keratin-10, claudin-1, ZO-1, and caveolin-1 were from Affinity Biosciences (Cincinnati, OH, USA). Antibodies against GAPDH and Horseradish peroxidase (HRP)-conjugated secondary antibodies against rabbit were provided by Santa Cruz Biotechnology (Danvers, MA, USA). Enhanced chemiluminescence reagent was obtained from Pierce Biotechnology (Rockford, IL, USA).

### Preparation of STCM-ATRA-NPs

HMSC membrane was prepared following published protocols [17]. Stem cells were lysed using hypotonic solution, followed by freeze–thaw disruption and differential centrifugation to get the purified stem cell membrane. To find the best way of combine the stem cell membrane with Isotretinoin (ATRA), we tried different methods, and adjusted the ratio between STCM and ATRA. ATRA was first dissolved in alcohol solution into 20 mg/mL, then added PBS solution into 4 mg/mL, 3 mg/mL, 2 mg/mL, 1 mg/mL, or 0.5 mg/mL. About 4 × 10^6^ stem cell derived stem cell membranes in PBS(1 mL) were used, subsequently, added 1 mL isotretinoin-alcohol/PBS solution, the mixture was placed on a constant temperature oscillator at 37 °C for 24 h and 48 h, avoiding light. Alternatively, using the Ultrasonic method, after thawing, 4 × 10^6^ stem cell derived stem cell membranes in PBS(1 mL) was placed in an ultrasound cleaner for 5 min, the isotretinoin-alcohol/PBS solution was mixed with the stem cell membrane solution and placed in the ultrasound cleaner for 10 min. The stem cell-coated isotretinoin was separated from uncoated isotretinoin by centrifugation at 15,000×*g* for 30 min at 4 °C, keeping the supernatant for UV mesurenment.

### Characterization of STCM-ATRA-NPs

#### Transmission electron microscopy

The morphology of the nanoparticles was observed by transmission electron microscopy (TEM; JEM-1230, JEOL, Tokyo, Japan). The samples were dispersed directly into bi-distilled water. A drop of the STCM-ATRA-NPs suspension was transferred to a 300-mesh carbon-coated copper grid. After staining with 2% (w/v) phosphotungstic acid solution and drying at room temperature, the sample was observed by TEM at 70 kV.

#### Determination of encapsulation efficiency of stem cell membrane-loaded isotretinoin

To calculate encapsulation efficiency, after extruded from a 200 nm filter membrane, the amount of uncoated isotretinoin was measured by UV absorbance spectrophotometrically at λ = 355 nm. The encapsulation efficiency was relative to the original drug added, applying the following equation: Encapsulation efficiency = total drug amount − unloaded drug amount/total drug.

### STCM-ATRA-NPs size analysis

The freshly prepared STCM-ATRA-NPs dispersion was diluted with double diluted water, and the nanoparticles were extruded from a 400 nm filter membrane. Following, a Dynamic Light Scattering particle size distribution analyzer (DLS, Brookhaven BI9000AT, New York, USA) was used to characterize the vesicle size and size distribution. The vesicle size range was set between 0.1 and 20 mm.

### Zeta potential of STCM-ATRA-NPs

Zeta potential, an indicator of stability of the STCM-ATRA-NPs dispersion, was determined using Malvern instruments (Osaka, Japan).

### Ultra-violet spectrophotometry of STCM-ATRA-NPs

An equal ATRA concentration of STCM-ATRA-NPs and STCM was sent for Ultra-violet spectrophotometry measurement at λ = 355 nm.

### In vitro release study

Protected from light, isotretinoin release from the stem cell membrane was performed using the Franz diffusion cell (RYJ-6B, HuangHai, Shanghai, China). These cells consisted of donor and receptor chambers separated by dialysis tubing with a molecular weight cut-off of 12,000–14,000 (Spectrum Medical Inc., Los Angeles, CA, USA). The receptor cell was filled with 50% alcohol/50% PBS, and 1.0 mL/0.1 mg/mL STCM-ATRA-NPs was added to the donor cell. Samples were removed from the side arm at 0.5, 24, 48, 72, 96, 120, 144, and 168 h, and an equal volume of blank solution was added to the receptor cell. Isotretinoin concentration in samples was measured by ultra-violet spectrophotometry at λ = 355 nm. Measurements were carried out in triplicate.

### Skin permeation test

Isotretinoin release was measured as described above. Briefly, a 1-month-old Yorkshire pig was sacrificed for its skin, and the subcutaneous fat was removed carefully to keep the stratum corneum intact. All animal experiments were performed in accordance with the guidelines for the Care and Use of Experimental Animals of Jilin University and were approved by the Animal Experiment Ethics Committee of Jilin University. Skin was kept at -80 °C before use. Further, skin was soaked in PBS for 1 h at 37 °C, and the skin was cut into small pieces (about 30 × 30 mm square samples) and placed between the receptor and donor cell. Additionally, 0.25 mg/mL ATRA (1 mL) and an equal weight of STCM-ATRA-NPs containing 0.25 mg/mL ATRA (1 mL) were added to the donor cell. An equal volume of stem cell membrane was added as the control group. The receptor cell was filled with 50% Isopropanol and 50% PBS without isotretinoin. Samples were taken from the receptor cell at 0.5, 1, 2, 4, 6, and 8 h at a volume of 0.5 mL, filtered, and quantified by HPLC (Acchrom S6000, Huapu Tec Inc., Beijing, China). An equal volume of receiving chamber solution without isotretinoin was added to the receptor cells. All studies were run at 37 °C, 100 rpm, avoiding light. Each experiment was repeated in triplicate.

### Skin retention test

The amount of drug retained in the skin samples was measured in permeation studies. After a skin penetration test, the skin was removed from the receptor cell, washed with PBS three times, and cleaned to remove any adhering formulation. Following, the skin was cut into small pieces, homogenized with 20 mL of chloroform: methanol at a volume ratio of 1:2, and vortexed for 10 min. After filtration using a 400 nm filter membrane, the drug content was measured by HPLC (Acchrom S6000).

### Observation of drug distribution and retention by fluorescence microscopy

Briefly, 1-month Yorkshire pig skin was cut into small pieces and set between the receiving and donor chamber of the Franz diffusion cell. The receptor cell was filled with 50% Isopropanol and 50% PBS without Isotretinoin. Following, PBS, 0.25 mg/mL ATRA, STCM, and an equal volume of STCM-ATRA-NPs containing 0.25 mg/mL ATRA (1 mL) were added to the donor chamber. Skin was treated for 8 h at 37 °C, 100 rpm, avoiding light. Skin tissue was fixed with 4% paraformaldehyde overnight and embedded in paraffin. Fluorescence microscopy was conducted to observe the fluorescence distribution, and ImageJ software was used to quantify fluorescence.

### Skin irritation test

Briefly, 12 New Zealand white rabbits were randomly assigned to four experimental groups: normal control, ATRA, STCM, and STCM-ATRA-NPs. PBS, 0.25 mg/mL ATRA cream, an equal volume of STCM, and an equal weight of ATRA-STCM-NPs containing 0.25 mg/mL ATRA were applied once to each group at a dose of 0.5 g. After 6 h, the test area was cleaned to observe visible changes. The scores were recorded (0–4) according to evaluation standards [25], with 0 indicating no erythema, 1 = slight erythema, 2 = moderate erythema, 3 = moderate-to-severe erythema, and 4 = severe erythema.

### Treatment effect in follicular hyperkeratosis model

Male New Zealand white rabbits (2–2.5 kg; Changchun Biological Products Co. LTD) were housed 1 per cage under a light/dark cycle of 12 h with free access to water and food continuously for 5 weeks. To establish the follicular hyperkeratosis model, 15 rabbits were randomly assigned into five groups: normal control (blank), hyperkeratosis + PBS (vehicle), ATRA, STCM, and STCM-ATRA-NPs. Further, 12 rabbits were given coal tar once a day on the ear tube (about 2 × 2 m^2^) for 14 days to establish the Kligman acne model. Based on established acne criteria, 0 indicates no erythema; 1 = slight erythema, comedo; 2 = moderate erythema, comedo; 3 = moderate-to-severe erythema, comedo; and 4 = severe erythema, comedo. Histological changes were evaluated by H&E staining and IHC staining.

### Treatment effect in hyper sebum production model

Male golden hamsters (6–8 weeks, 80–120 g) were used to establish a hyper sebum production model. Hair was removed carefully to expose the two sides of the sebaceous glands, and the hamsters were randomly divided into four groups: normal control (blank), ATRA, STCM, and STCM-ATRA-NPs. Formulations were given once daily, and the animals were sacrificed on day 14. Histological changes were evaluated by H&E staining.

### Scanning electron microscopy and Fourier infrared spectrum analysis

Briefly, 1-month-old Yorkshire pig skin was processed in the Franz diffusion cell, as described above. The receptor cell was filled with 50% Isopropanol and 50% PBS without Isotretinoin. Further, PBS, 0.25 mg/mL ATRA, STCM, and an equal volume of STCM-ATRA-NPs containing 0.25 mg/mL ATRA (1 mL) were added to the donor chamber. Skin was treated for 8 h at 37 °C, 100 rpm, avoiding light. For SEM analysis, the skin was fixed with glutaraldehyde for 24 h, freeze-dried for 48 h, and observed using a scanning electron microscope (Carl Zeiss Microscopy GmbH, Oberkochen, Germany) to assess changes in the Horney layer of the skin layer at 200× and 900× magnification. For Fourier infrared (FTIR) spectral analysis, the skin was removed from the freeze-drying machine after 48 h, ground with potassium bromide using an agate mortar, and analyzed at resolution: 2 cm^−1^, scanning times: 100, and scan range: 650–4000 cm^−1^ to assess changes in keratin.

### Western blotting

Briefly, 1-month-old Yorkshire pig skin was processed in the Franz diffusion cell, as described above. After treatment for 8 h at 37 °C, 100 rpm, avoiding light, the skin was removed and ground in liquid nitrogen for protein extraction. Ice-cold lysis buffer was used to treat skin tissue samples, and the relative concentrations were determined by BCA protein kit. The supernatants of the cell lysates were then separated by 12% SDS-PAGE gels and transferred onto PVDF membranes, which were subsequently blocked with 5% no-fat milk. Finally, the membranes were reacted with primary antibodies for 12 h, followed by keratin-10, claudin-1, ZO-1, caveolin-1 and clathrin heavy chain secondary antibodies.

### Statistical analysis

All data are given as mean ± standard error (SE). Statistical analyses were performed using two-sample Student’s *t* tests and ANOVAs, followed by an LSD post hoc test. *P* < 0.05 was considered statistically significant.

## Results

### Synthesis and characterization of ATRA-STCM-NPs

**Fig. 1:**
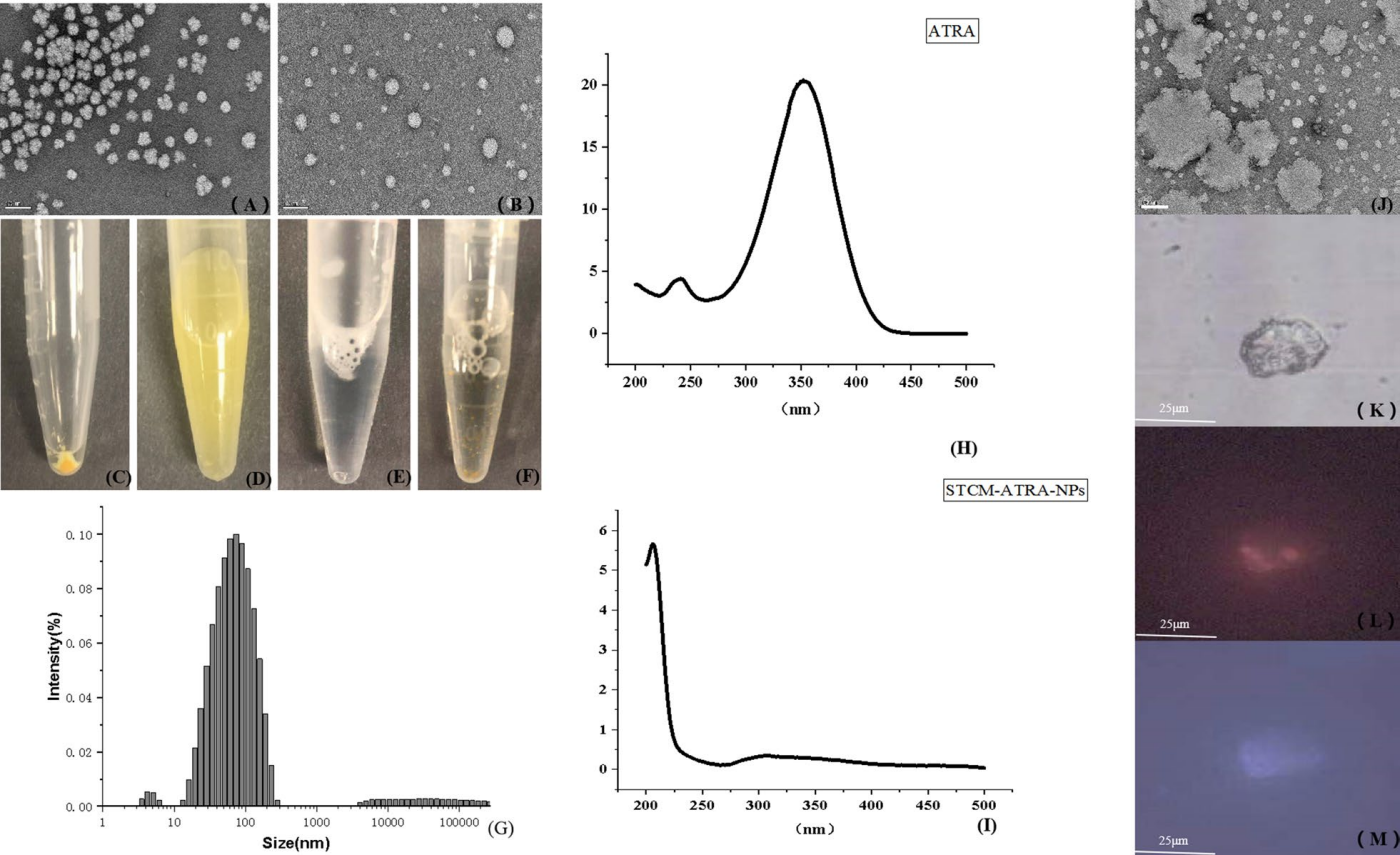
Characterization of STCM-ATRA-NPs. **a** Morphology of stem cell membrane by TEM. **b** Morphology of STCM-ATRA-NPs, **c** freshly prepared STCM-ATRA-NPs, **d** STCM-ATRA-NPs in PBS, **e** stem cell membrane in PBS, and **f** isotretinoin in PBS. **g** Size of STCM-ATRA-NPs measured by DLS and **h** ATRA and **i** STCM-ATRA-NPs measured by ultra violet spectrum. **j** Morphology of oscillator-generated STCM-ATRA-NPs by TEM. **k** Morphology of oscillator-generated STCM-ATRA-NPs by microscope. **l** Morphology of oscillator-generated STCM-ATRA-NPs by fluorescence microscopy under blue fluorescence and **m** under ultraviolet

STCM-coated isotretinoin using different methods and components showed different encapsulate efficiencies, and the results are shown in Table 1. The standard curve of Isotretinoin is y = 183.22x − 5.5199, R^2^ = 0.9996 (Isotretinoin as standard dissolved in a 50%PBS/50% alcohol).The ultrasonic approach appeared to be much better than the oscillator method. Oscillator-generated stem cell membrane-coated ATRA were shown as irregular and huge particles in TEM (Fig. 1j) and microscope (Fig. 1k) observation. For further observation, stem cell membrane were labeled with fluorescence probe (PKH-26, Sigma-Aldrich, shanghai, China), it could seen that the fluorescence of cell membrane (Fig. 1l) coincided with that of ATRA (Fig. 1m). In TEM observations of the morphology of ultrasonic-generated STCM-ATRA-NPs, homogeneous, rounded nanoparticles were observed. As such, the ultrasonic method at the ratio of 4 × 10^6^ stem cells derived membranes and 3 mg/mL ATRA(1 mL) was selected to generate STCM-ATRA-NPs for subsequent analysis.

**Table 1:** Encapsulation efficiency of STCM-ATRA-NPs

Serial number	Concentration of isotretinoin (mg/mL)	Encapsulation method and time	Encapsulation efficiency (%)
1	4	Ultrasonic	80.07
2	3	Ultrasonic	88.59
3	2	Ultrasonic	78.98
4	1	Ultrasonic	42.07
5	4	Oscillator (24 h)	30.04
6	3	Oscillator (24 h)	28.54
7	2	Oscillator (24 h)	27.33
8	1	Oscillator (24 h)	15.42
9	4	Oscillator (48 h)	32.49
10	3	Oscillator (48 h)	31.32
11	2	Oscillator (48 h)	27.54
12	1	Oscillator (48 h)	16.08

Isotretinoin appeared to be insoluble in water, with yellow particles deposited at the bottom of the tube (Fig. 1f). However, isotretinoin dissolved in ethanol, becoming a transparent yellow liquid. Stem cell membrane dissolved in water appeared as a colorless and transparent liquid. After mixing ATRA-alcohol and STCM solutions together by the ultrasonic method, followed by centrifugation at 15,000×*g*, the yellow precipitate (STCM-ATRA-NPs) dissolved in water, forming a yellow suspension. The DLS results showed that the mean diameter of nanoparticles is 56.9 nm (Std deviation = 0.221), which is consistent with the TEM results (Fig. 1g). To confirm the new nanoparticle, using the UV spectrum to test the unique peak of ATRA, STCM-ATRA-NPs did not show the unique absorption peak, which may be due to embedding of isotretinoin molecules in the lipid bilayer of the stem cell membrane. The zeta potential of STCM-ATRA-NPs is − 34.9 mV, STCM-ATRA-NPs showed great stability.

### Sustained release of nanoparticles in in vitro release study

**Fig. 2:**
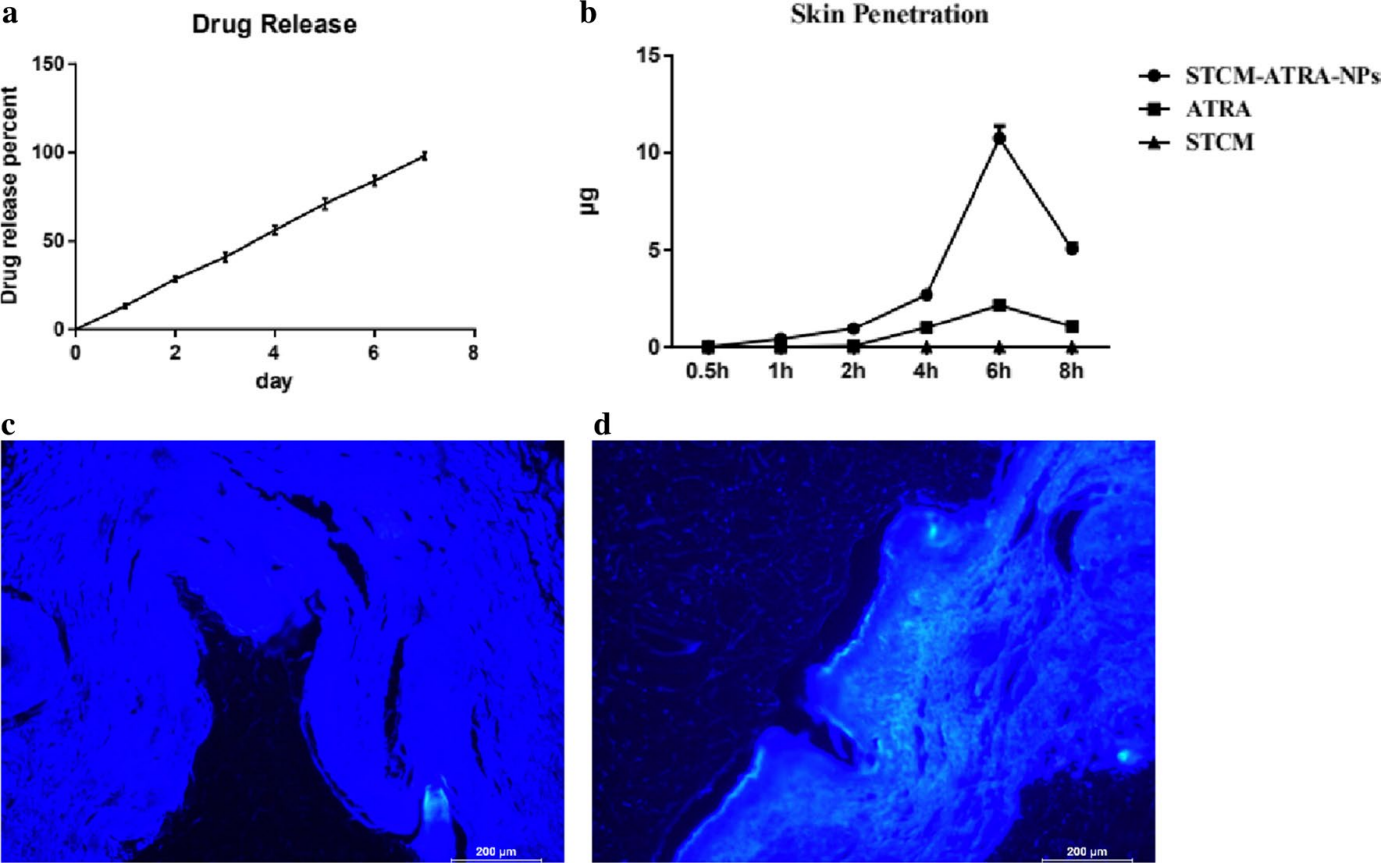
Drug loading characters of STCM-ATRA-NPs. **a** Controlled release of ATRA from STCM-ATRA-NPs, followed daily at 37 °C in a hydroalcoholic solution, up to 7 days. **b** Penetration of ATRA and STCM-ATRA-NPs through the pig dermis, monitored over 8 h. Data shown are mean ± S.D., n = 3. Franz diffusion cell of **c** ATRA and **d** STCM-ATRA-NPs for 8 h at 37 °C and 100 rpm. STCM-ATRA-NPs at 8 h showed more uniform distribution and accumulation compared to ATRA

Figure 2a shows the in vitro release profiles of STCM-ATRA-NPs, demonstrating sustained release function. The overall release of ATRA from the STCM-ATRA-NPs was very slow, which may provide a better therapeutic effect. The results showed that ATRA can be released completely from the stem cell membrane at 168 h. Isotretinoin was shown to be very unstable and easily decomposed, while ATRA released from the stem cell membrane showed great stability.

### Greater transdermal ability of nanoparticles in skin permeation test

Figure 2b shows that STCM-ATRA-NPs demonstrated greater transdermal ability than free ATRA. Further, stem cell membrane-coated isotretinoin showed faster permeation across the skin than the Isotretinoin.

### Higher skin retention of nanoparticles

Skin retention of ATRA in skin was detected by HPLC measurement, STCM-ATRA-NPs group is 0.45 ± 0.13 μg (n = 3), while ATRA group is 3.03 ± 0.17 μg (n = 3). HPLC results showed that STCM-ATRA-NPs were retained in skin much less than ATRA. Both STCM-ATRA-NPs and ATRA can show white fluorescence of under UV excitation (365 nm). From observation of fluorescence microscope, fluorescence of STCM-ATRA-NPs (Fig. 2d) appeared much stronger than free ATRA (Fig. 2c), and STCM-ATRA-NPs were distributed more uniformly than free ATRA. The opposite results may because STCM-ATRA-NPs barely have no absorbance at λ = 355 nm, it can not be measured by HPLC, it may indicated ATRA were slowly release from STCM-ATRA-NPs in skin.

### Reduced skin irritation by nanoparticles

**Fig. 3:**
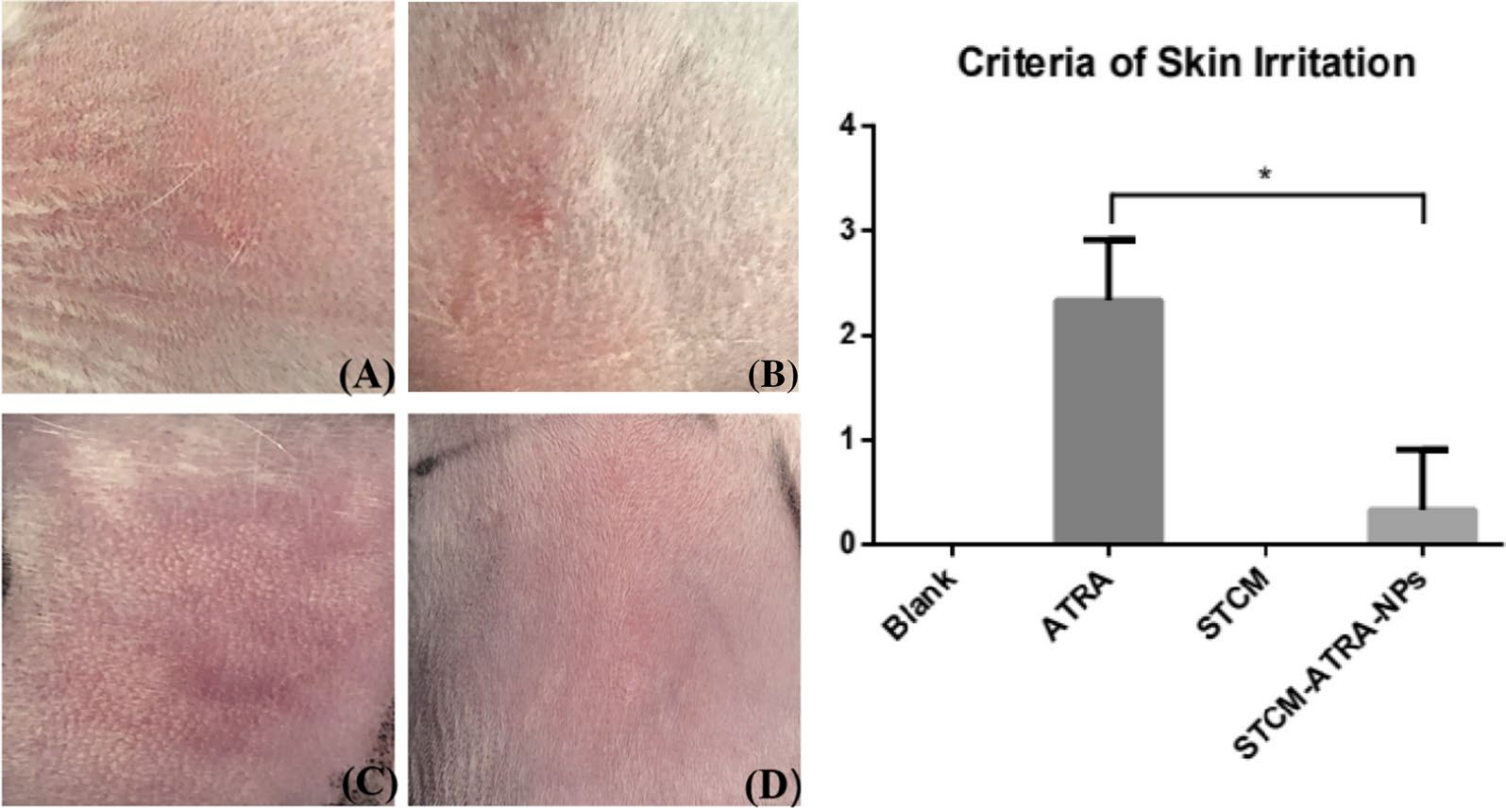
Skin irritation by STCM-ATRA-NPs. Skin application of **a** PBS, **b** STCM, **c** ATRA, or **d** STCM-ATRA-NPs for 6 h. **e** Criteria of skin irritation. Data shown are mean ± S.D., n = 3

Figure 3 shows that after 6 h, the STCM-ATRA-NPs group showed no erythema to very slight erythema, while isotretinoin caused slight to moderate erythema (P = 0.016). Additionally, the STCM group showed no erythema.

### Greater treatment efficiency in hyperkeratinization model

**Fig. 4:**
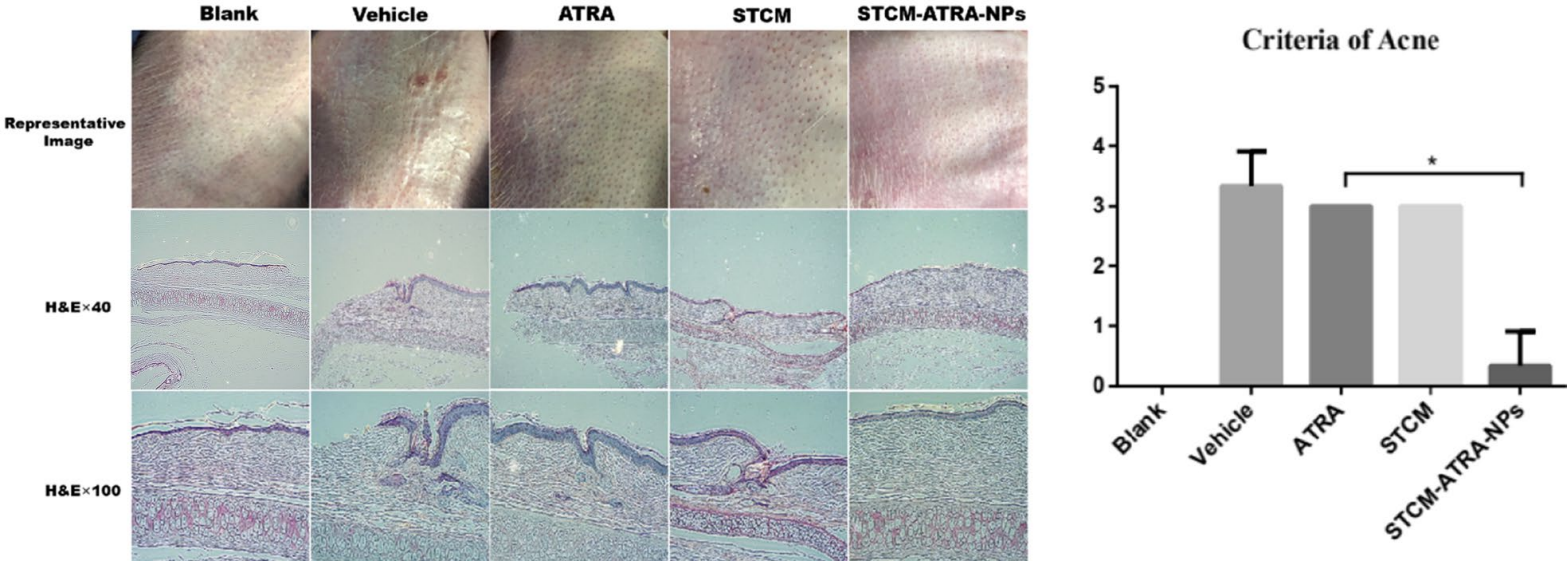
Treatment efficiency on hyper keratinization model by H&E staining. Representative image, HE staining (at ×40 and ×200 magnification), and acne criteria of hyper keratinization model

Figure 4 shows that STCM-ATRA-NPs had a great therapeutic effect in a hyperkeratinization model. Apparently, STCM-ATRA-NPs reduced comedo. After 14-day treatment, the STCM-ATRA-NPs group had no comedo or slight comedo. ATRA and STCM groups showed slight-to-moderate comedo (P = 0.116).

**Fig. 5:**
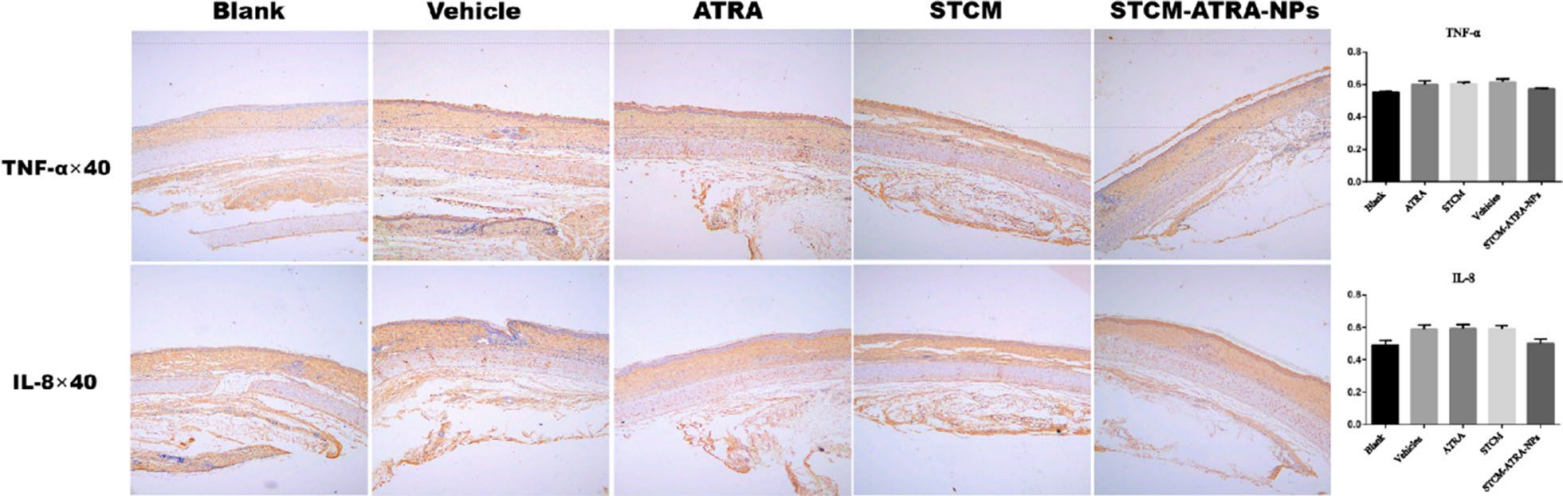
Treatment efficiency on hyper keratinization model by IHC staining. Representative images and quantification of IL-8 and TNF-α IHC staining of hyper keratinization acne model. Data shown are mean ± S.D., n = 3

**Fig. 6:**
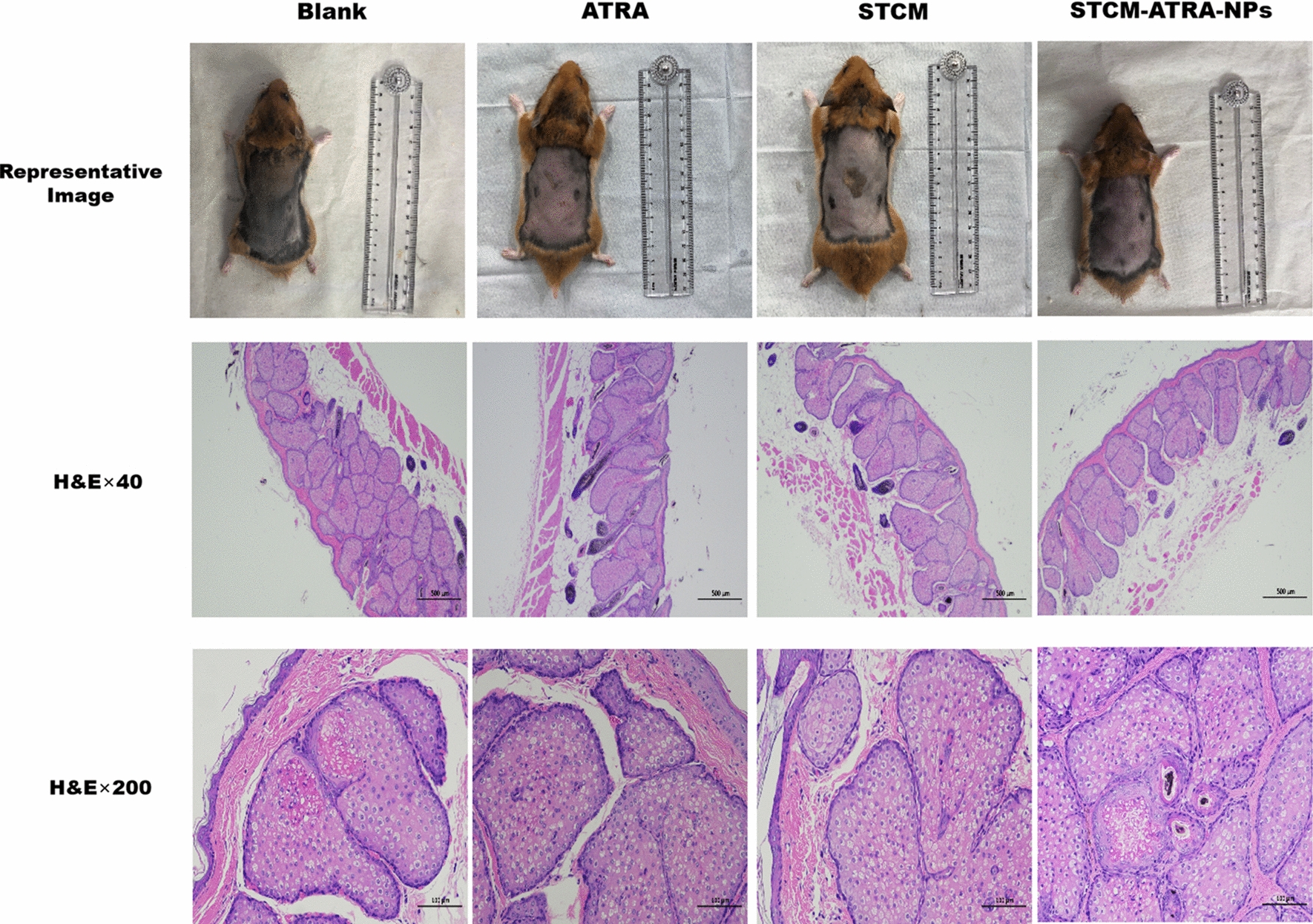
Treatment efficiency on hyper sebum production model. Representative image and HE staining (at ×40 and ×200 magnification) of hyper sebum production acne model. Data shown are mean ± S.D., n = 3

From H&E staining results (Fig. 4), STCM-ATRA-NPs caused no follicular keratotic plugging, indicating reduced hyperkeratinization. However, there were no significant differences in IL-8 and TNF-α expression, two major inflammatory factors in acne, between STCM-ATRA-NPs and ATRA groups (Fig. 5), indicating STCM-ATRA-NPs may not effectively impact inflammation. Further, no obvious change in hyper sebum production was observed, as measured by sebaceous glands volume and the number of leaf layers of sebaceous glands (Fig. 6).

### Altered morphology of the skin Horney layer

**Fig. 7:**
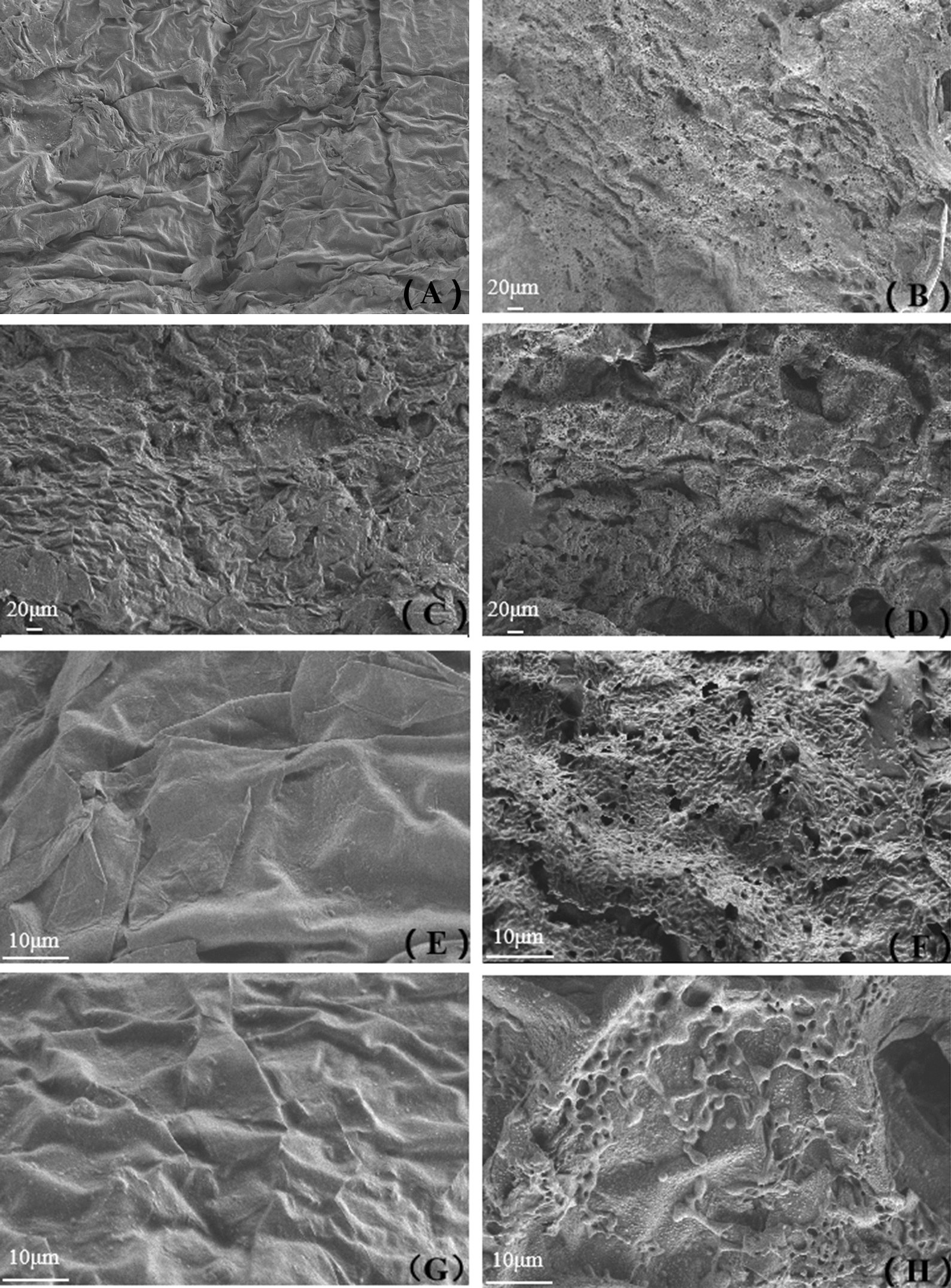
Morphology of the skin Horney layer. Skin treated with **a** PBS, **b** STCM, **c** ATRA, or **d** STCM-ATRA-NPs for 6 h, at ×200 magnification. Skin treated with **e** PBS, **f** STCM, **g** ATRA, or **h** STCM-ATRA-NPs for 8 h, at ×900 magnification

Figure 7 shows that STCM-ATRA-NPs could penetrate the main skin barrier. The effects of the stem cell membrane and STCM-ATRA-NPs on the structure of the corneum were observed by SEM. Compared with the control group, the stem cell membrane and STCM-ATRA-NPs created a porous structure, destroying its original smooth surface.

### Altered expression of endocytosis-related proteins

**Fig. 8:**
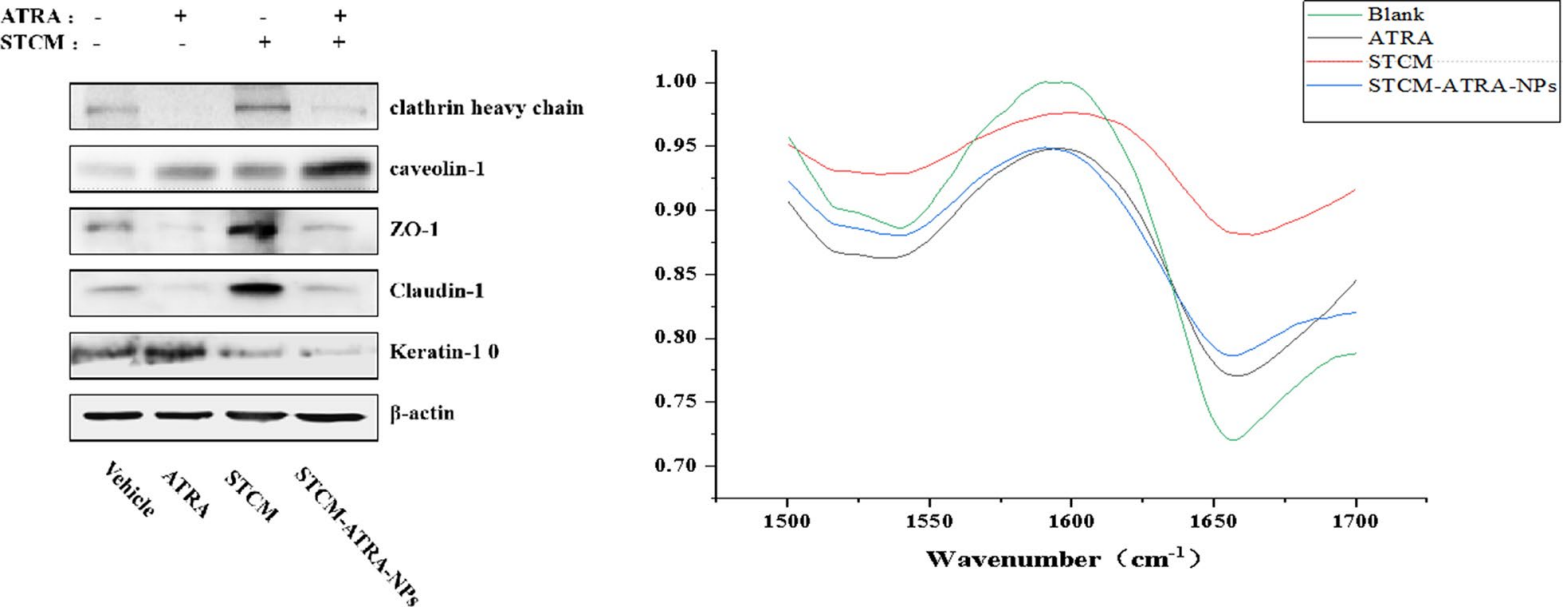
Mechanism of STCM-ATRA-NP transdermal enhancement. **a** The levels of endocytosis-related proteins, tight junction-related proteins, and the main protein of the LC layer treated by PBS, ATRA, STCM, and STCM-ATRA-NPs, in a Franz diffusion cell for 8 h, as measured by western blot. **b** The change in keratin as analyzed by Fourier infrared spectral analysis

Compared to the ATRA group, the STCM-ATRA-NPs group showed reduced expression of the keratin 10 proteins. By contrast, endocytosis-related proteins clathrin heavy chain and caveolin-1 were increased, which may indicate that STCM-ATRA-NPs permeated through the skin via an endocytosis pathway and removed the barrier of the SC and tight junctions [26, 27] (Fig. 8a). Our results showed that caveolin-1 was increased, suggesting that the STCM-ATRA-NPs transdermal behavior is dependent on caveolin-1 mediated endocytosis [28].

### Altered keratin structure in the skin

FITR analysis indicated disruption of keratin in skin. The special absorption peak of keratin in the SC was the stretching vibration of amides I (1600–1700 cm^−1^) and amides II (1500–1600 cm^−1^) (Fig. 8b). However, the unique peak of amides I and amides II was changed slightly, indicating that the alpha helix of keratin in the skin was destroyed, and the structure of keratin in the skin had changed.

## Discussion

Overall, our study established a new approach of coating isotretinoin in a stem cell membrane, discovering the function of endocytosis-related keratin and tight junction proteins in the nanoparticle’s transdermal pathway. Most isotretinoin drug carriers are liposomes and generated through chemical synthesis, which are relatively toxic. This study describes the natural cell membrane as a drug delivery system. STCM-ATRA-NPs are small (less than 100 nm), stable nanoparticles. Isotretinoin is easy to oxidize and photolyze [29], but it shows great stability when combined with the stem cell membrane. Compared with the traditional isotretinoin gel, the stem cell membrane can help to preserve isotretinoin, effectively preventing isotretinoin decomposition. Further, the stem cell membrane can control release up to 7 days, which is an ideal feature of a great drug carrier. We established two acne models to evaluate the effect of STCM-ATRA-NPs on different acne pathogeneses. The hyper keratinization model showed an acceptable result, in which it worked effectively on hyperkeratinization. However, it showed no effect on hyper sebum production, which may be the result of the impact of STCM-ATRA-NPs on keratin and tight junctions. Yet, they may not permeate through sebaceous glands tissue completely, which requires further investigation.

The skin retention test and distribution observed by fluorescence microscopy demonstrated that STCM-ATRA-NPs can facilitate isotretinoin retention in skin. By contrast, the skin retention study showed the opposite results, suggesting that isotretinoin may not have been thoroughly released from STCM-ATRA-NPs, which may continue over several days. Further, the STCM-ATRA-NPs distribution results showed uniformity in density distribution, which may reduce side effects and provide better treatment.

**Fig. 9:**
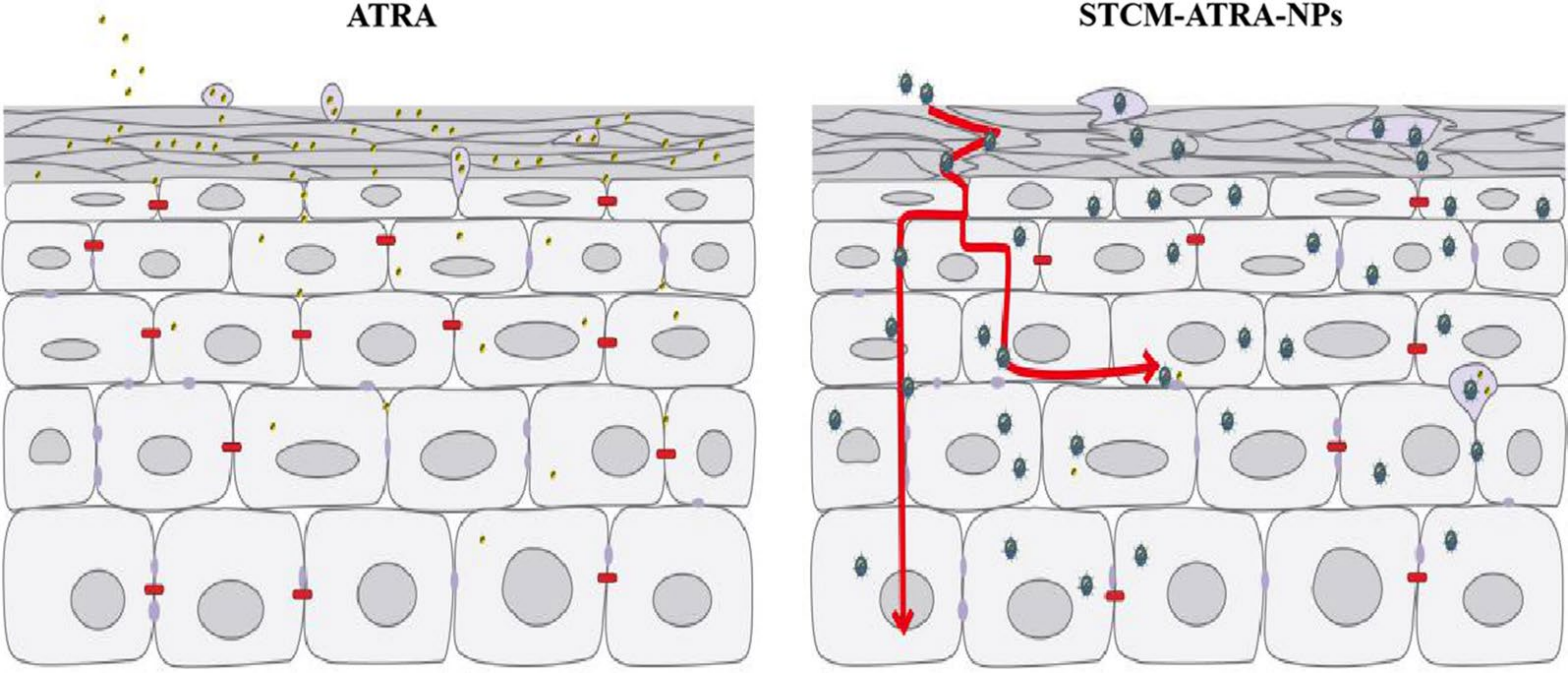
Proposed mechanism of STCM-ATRA-NP transdermal ability

The skin permeation study indicated that STCM-ATRA-NPs had great transdermal ability. Moreover, the western blot, SEM, and FTIR observations indicated the potential mechanism of STCM-ATRA-NPs improvement of transdermal activity. Some studies suggest that drug carriers transform to permeate the SC layer of the skin [29, 30], while others have shown that liposomes as drug carrier can impact keratin to enhance skin penetration [31, 32]. In our study, western blot, FTIR, and SEM findings all demonstrated that keratin was disrupted and decreased in the skin. Since the SC layer had some porous structures, STCM-ATRA-NPs may enter the SC layer through these pores, which is much faster than transformation. Further, tight junction proteins were decreased, while the functional proteins of endocytosis were increased. By contrast, after sealed with ATRA, keratin content increased, the endocytosis protein caveolin-1 slightly increased, but the endocytosis protein clathrin heavy chain was significantly decreased. Further, the tight junction protein was strongly increased with the stem cell membrane, which is in contrast to treatment by STCM-ATRA-NPs, and both the clathrin heavy chain and caveolin-1 protein were increased, indicating that the stem cell membrane can enter cells through two endocytic mechanisms, while STCM-ATRA-NPs can enter mainly through caveolin-1 protein-related pathways. Bioactivity of STCM-ATRA-NPs differs from the stem cell membrane, but the reason and mechanism still need further investigation. The different tendency of stem cell membrane and STCM-ATRA-NPs is related to the interact between tight junctions and the corneum layer. However, the mechanism by which the stem cell membrane interacts in this process still needs to be further explored. The tight junction and LC layer, as the two main barriers of transdermal delivery, are believed to interact with each other and alter lipid organization [33]. Based on our findings, we hypothesize that the LC layer, tight junction, and endocytosis all play an important part in the mechanism of transdermal delivery and can interact with each other (Fig. 9). However, this mechanism still needs further study.

## Conclusion

In conclusion, the STCM-ATRA-NPs demonstrated control isotretinoin release, reduced side effects, and efficiently permeating through the skin, it also showed significant therapeutic efficacy. We further demonstrated that the nanoparticles could enhance the transdermal efficacy of isotretinoin by reducing the effect of keratin and tight junction proteins. Further, nanoparticles enhanced endocytosis, thereby promoting drug penetration and absorption into the skin.

## Data Availability

Data sharing not applicable to this article as no datasets were generated or analyzed during the current study.

## Abbreviations

ATRA: Isotretinoin
BCA: Bicinchoninic acid
BSA: Bovine serum albumin
DLS: Dynamic light scattering
FBS: Fetal bovine serum
FTIR: Fourier transform infrared
H&E: Hematoxylin and eosin
hMSC: Human umbilical cord mesenchymal stem cell
HPLC: High-performance liquid chromatography
HRP: Horseradish peroxidase
IHC: Immunohistochemistry
PBS: Phosphate buffered saline
PVDF: Polyvinylidene fluoride
SC: Stratum corneum
STCM: Stem cell membrane
STCM-ATRA-NPs: Stem cell membrane-coated isotretinoin nanoparticles
SEM: Scanning electron
TEM: Transmission electron microscope
