# Correction to: Stem cell membrane-coated isotretinoin for acne treatment

**DOI:** 10.1186/s12951-021-00954-w

**Published:** 2021-08-06

**Authors:** Shiyi Wang, Rihua Jiang, Tianqi Meng, Fuqiang Zhang, Jing Li, Yongri Jin, JeungHoon Lee, Mingji Zhu, Jinlan Jiang

**Affiliations:** 1 Department of Dermatology, China-Japan Union Hospital of Jilin University, Changchun, Jilin China; 2 Scientific Research Center, China-Japan Union Hospital of Jilin University, Changchun, Jilin China; 3 College of Chemistry, Jilin University, Changchun, Jilin China; 4 Department of Dermatology, School of Medicine, Chungnam National University, Daejeon, Republic of Korea

## Correction to: J Nanobiotechnol (2020) 18:106 10.1186/s12951-020-00664-9

Following publication of our article [1] the authors found that some of the images in the publication had been misused, so that three images presented at high magnification did not correspond to those at low magnification. The correct images for Figure 3b, Figure 4 (Treatment efficiency on hyper keratinization model by H&E staining—Group Blank H&E × 40) and Figure 7a are shown below. These errors do not affect the conclusions of the work. The authors apologize for these errors and any inconvenience caused.

Original image
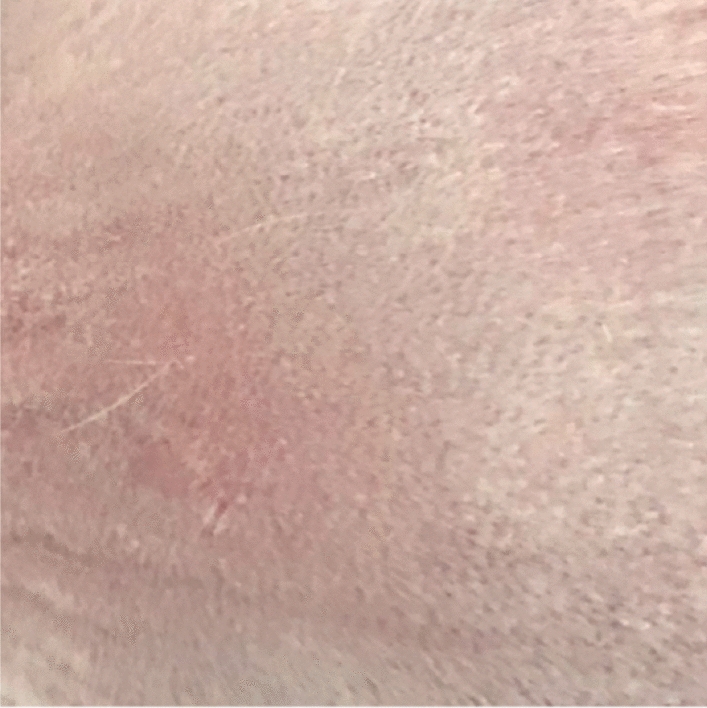


Change to
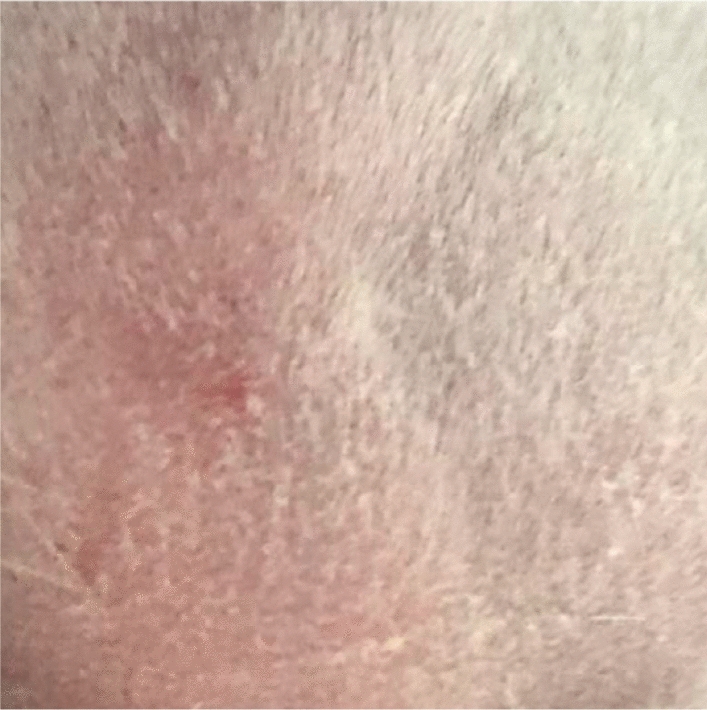


Figure 4 Treatment efficiency on hyper keratinization model by H&E staining.—Group Blank H&E × 40

Original image
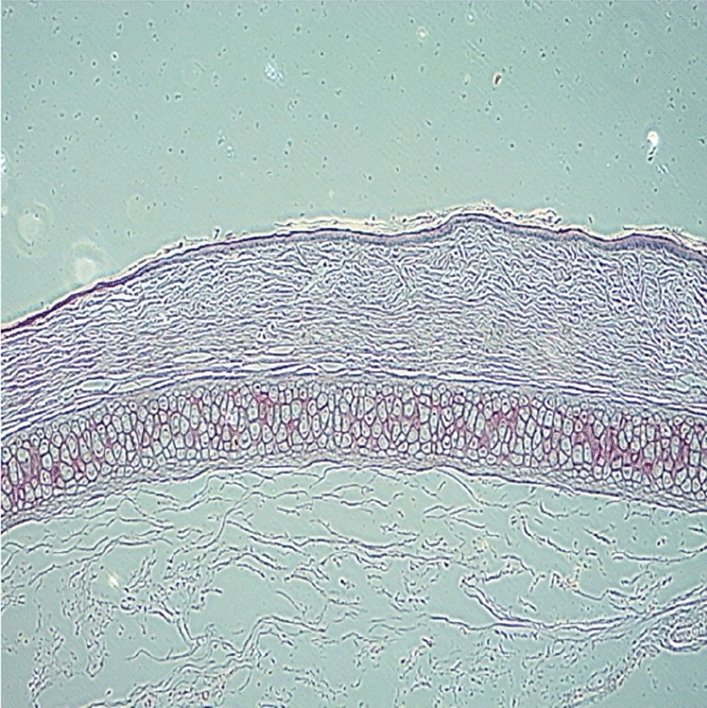


Change to
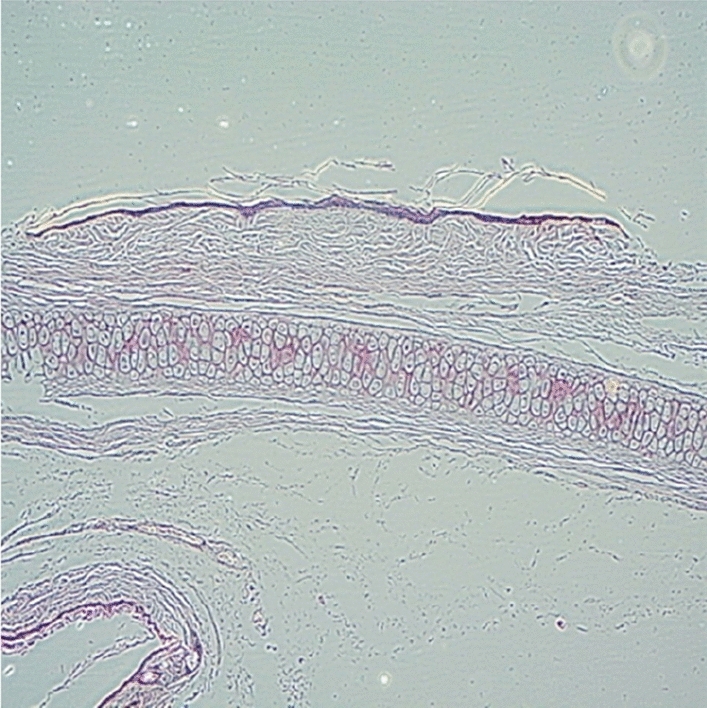


Figure 7 Morphology of the skin Horney layer.—7a Skin treated with PBS

Original image
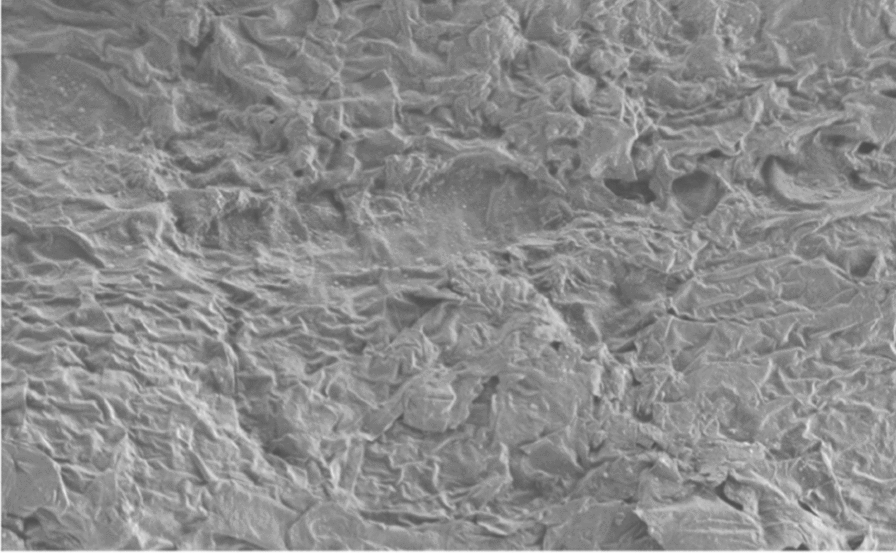


Change to
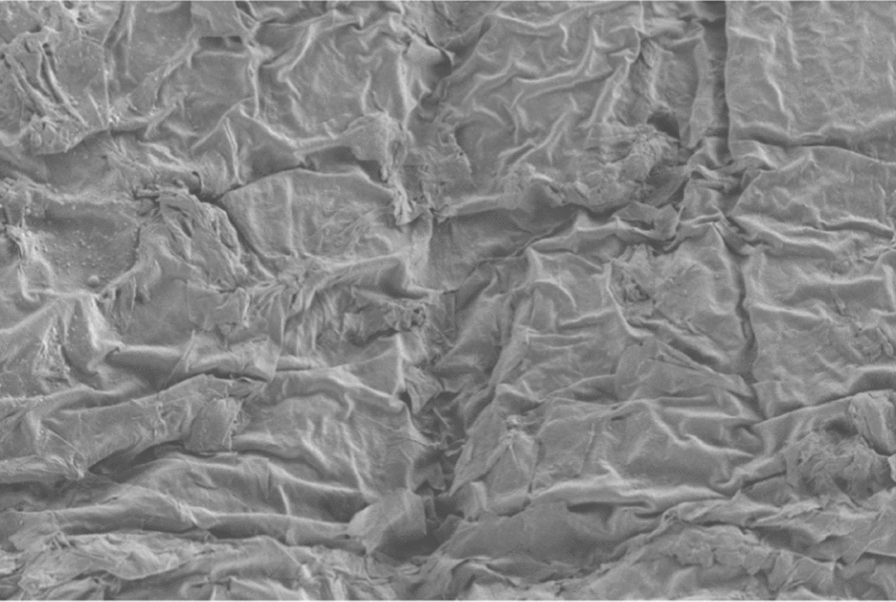


Figure 3 Skin irritation—3b Skin application of STCM

The original article has been revised.
